# Multistability in the epithelial-mesenchymal transition network

**DOI:** 10.1186/s12859-020-3413-1

**Published:** 2020-02-24

**Authors:** Ying Xin, Bree Cummins, Tomáš Gedeon

**Affiliations:** 1 Department of Ophthalmology (Wilmer Eye Institute), Johns Hopkins University School of Medicine, Baltimore, USA; 2 Department of Mathematical Sciences, Montana State University, Bozeman, USA

**Keywords:** Epithelial-mesenchymal transition, Multistability, Network models

## Abstract

**Background:**

The transitions between epithelial (E) and mesenchymal (M) cell phenotypes are essential in many biological processes like tissue development and cancer metastasis. Previous studies, both modeling and experimental, suggested that in addition to E and M states, the network responsible for these phenotypes exhibits intermediate phenotypes between E and M states. The number and importance of such states is subject to intense discussion in the epithelial-mesenchymal transition (EMT) community.

**Results:**

Previous modeling efforts used traditional bifurcation analysis to explore the number of the steady states that correspond to E, M and intermediate states by varying one or two parameters at a time. Since the system has dozens of parameters that are largely unknown, it remains a challenging problem to fully describe the potential set of states and their relationship across all parameters. We use the computational tool DSGRN (Dynamic Signatures Generated by Regulatory Networks) to explore the intermediate states of an EMT model network by computing summaries of the dynamics across all of parameter space. We find that the only attractors in the system are equilibria, that E and M states dominate across parameter space, but that bistability and multistability are common. Even at extreme levels of some of the known inducers of the transition, there is a certain proportion of the parameter space at which an E or an M state co-exists with other stable steady states.

**Conclusions:**

Our results suggest that the multistability is broadly present in the EMT network across parameters and thus response of cells to signals may strongly depend on the particular cell line and genetic background.

## Background

The epithelial-to-mesenchymal transition (EMT) and mesenchymal-to-epithelial transition (MET) are essential processes of cellular plasticity. This plasticity manifests itself in embryonic development [1, 2] and wound healing [3, 4], but it is also of great interest for its role in carcinoma metastasis [5]. Activation of the EMT program leads to a tumor-initiating state sometimes termed cancer stem cell (CSC) [6, 7]. In addition, the EMT program modulates the immune response of the organism [8, 9] and negatively affects immunotherapy.

The epithelial phenotype is characterized by apical-basal polarity and tight cell adhesion to the other cells in the tissue. The hallmarks of the transition to the mesenchymal phenotype are the loss of adhesion, gain of motility, and acquisition of invasive capabilities. In EMT, cells may not complete the transition to the fully mesenchymal phenotype, but acquire one of possibly many partially epithelial and partially mesenchymal states (E/M states or intermediate states). At least one intermediate state has been experimentally documented in several tissues [10–12]. These tissues exhibit the presence of biomarkers for both mesenchymal and epithelial states on the level of a single cell [11–14], observed in lung cancer [15] as well as in metastatic brain tumors [16]. Therefore an E/M state is not just a mixture of cells of both phenotypes.

There is evidence that cells in an intermediate state exhibit a different phenotype. They retain some adhesiveness to their neighbors and seem to migrate in clusters. This intermediate phenotype has consequences for cancer prognosis; when cells migrate in the intermediate phenotype it usually indicates poor prognosis. While it is clear that initiation of the EMT program plays a key role in initiation of metastasis, the reverse MET program occurs during the last step of the process, colonization of the new niche, adapted to the micro-environment of the invaded tissue. Why certain cells succeed in colonization, while the majority probably do not, is not clear. Some cells may fortuitously develop adaptive programs while still in the primary sites and may maintain them during colonization; the diversity of the E/M states and cellular background may play a decisive role in colonization success [5, 17].

It is therefore important not only to characterize the intermediate E/M states, and the pattern of activation that leads to each of them, but also the pattern of activity of other elements of the network in each of these states. It may be that the activity of genes not directly connected to known biomarkers is decisive in the success or failure of colonization of a new tissue. Furthermore, one of the potential treatments for EMT-induced cellular motility and carcinoma metastasis is induction of MET. Apart from the possibility that this treatment would make colonization easier for the cells that have already migrated, it is not clear if the final state after the treatment would indeed be the epithelial state or some form of intermediate state due to hysteresis in nonlinear systems.

Because of the clinical significance, there is great interest in understanding the networks that are responsible for this phenotypic transition and to characterize the intermediate E/M states [17]. It has been suggested that these states are only metastable [3, 18] and cannot be maintained in the long term. On the other hand, extensive modeling work has shown that an E/M state is represented by a stable state of a network [11, 12, 19–21]. These papers analyzed the contributions of miR34-Snail1 and miR200-Zeb1 bistable modules [19, 20] to EMT and MET processes and the contribution of Ovol2 and GRHL2 to the existence and robustness of this state [21], as well as the extent to which this intermediate state is connected to the development of stemness, a cellular trait associated with increased invasiveness [6, 22, 23]. Hong et al. [11] modeled a network that includes Ovol2, Zeb1, Snail1, miR34a, miR200 and TGF *β* depicted in Fig. 2a. They show in their model, and also find experimental evidence, that there exists not one, but two intermediate states *I*_1_ and *I*_2_. Using ODE models they show that both states are sensitive to Ovol2 levels and overexpression of Ovol2 leads to a transition of the system to the epithelial state. Similarly, a high level of TGF *β* induces the mesenchymal state, while a low dose of TGF *β* induces the appearance of coexisting populations of *I*_2_ and *M* states.

**Fig. 1:**
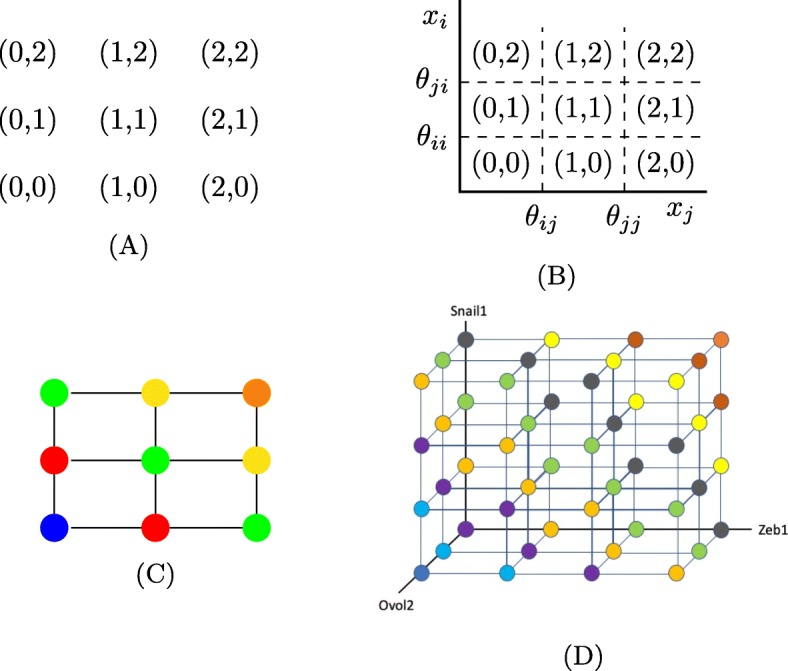
State transition graph representation of dynamics. **a** The nodes of all STGs (the set *S*) in a network with two genes that both regulate themselves and each other. **b** The corresponding embedding into two-dimensional phase space. **c** The discrete grid construction of phase space with constant Hamming distances from the extreme corners represented by color. **d** The projection of the states in the six-dimensional phase space of the EMT network to 3 dimensions corresponding to Zeb1, Snail1 and Ovol2. The colors divide this 3D cube into nine diagonals, each of which has a fixed Hamming distance from the extreme values representing E and M states. The E state is in the lower left-hand corner in the front, and the M state is in the upper right-hand corner in the back

**Fig. 2:**
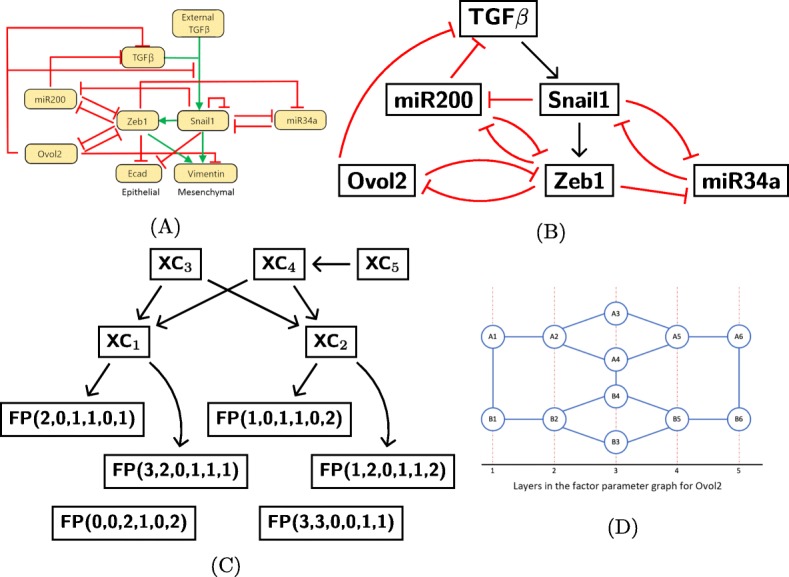
Parameter graph representation of the parameter space. **a** The EMT network from [11]. **b** The EMT network that we use for the analysis in this manuscript. **c** An example of the many possible Morse graphs for the network in (**b**). **d** The factor parameter graph for Ovol2. Each node represents one way in which the inputs of Ovol2 are integrated and affect the downstream nodes of Ovol2. Each node is characterized by the corresponding inequalities given in (1). Nodes colored in red are associated to essential parameters

Mathematical models based on ODEs of complex networks like the EMT network face significant challenges. The simulation of differential equations requires precise parameterization and initial conditions; these are difficult to ascertain in cellular systems. Bifurcation analysis, such as that presented in [11, 21], allows one or two parameters to vary at a time, but the other parameters (often numbering in the dozens) need to be fixed. Many important insights were obtained using careful bounds on parameters and sensitivity analysis, but the challenges of interpretability and generality of the results remain. To address these challenges, Huang et.al. [24] developed a computational method RACIPE that samples random kinetic models corresponding to a fixed circuit topology, and then uses statistical tools to gain insight into properties of the circuit that are robust with respect to choices of kinetic parameters. An alternative approach to study the behavior of a large EMT network without knowledge of the kinetic parameters is to study a Boolean network model [25]. This study employed energy associated to glassy states to study the robustness and number of steady states associated to E, M, and E/M states.

In this paper we present an alternative analysis of the complex EMT network based on the software package DSGRN (Dynamic Signatures of Gene Regulatory Networks) [26–31]. DSGRN uses a continuous description of network dynamics that lends itself to a discrete and exhaustive description of all the ways in which the network can function, specified only by inequalities between network parameters. For each such set of parameters DSGRN characterizes network dynamics in terms of *a state transition graph* that can be reduced to an acyclic graph called a *Morse graph*. A state transition graph can be interpreted as an asynchronous update of a corresponding monotone Boolean map. Therefore DSGRN can be viewed as examination of a collection of monotone Boolean maps consistent with the network topology. On the other hand, the underlying continuous time description of dynamics allows one to relate DSGRN parameters to parameters of Hill function kinetic models that are sampled in the statistical approach of Huang et.al. [24] (see Remark 1 of “Methods” section).

The leaves of a Morse graph represent invariant sets of the system, including steady states. Results of DSGRN computation allows us to find representatives of epithelial, mesenchymal, and intermediate states. Rather than computing bifurcation diagrams for one or two varying parameters, our results describe possible dynamics at all combinations of parameters. Our results are coarse; as explained in “Methods” section, we assume each edge in the network has a threshold and the effect on the downstream gene has two levels, low and high, all of which are real-valued parameters. However, the methodology behind DSGRN allows us to decompose parameter space into a finite (but very large) number of parameter domains over each of which the Morse graph is constant. We represent this decomposition as *parameter nodes* in a *parameter graph*, where each node is associated to a Morse graph. This computational output is then interrogated to find equilibria, other types of attractors, bistability, and multistability.

Our first result is that in all parameter nodes the only attractors in the system are steady states. We also detect the presence of potential oscillatory behavior, but it is always unstable within our framework.

While we compute the entire collection of dynamics across parameter space, we present them as four separate projections over parameters that represent Ovol2, Snail1, TGF *β*, and Zeb1 expression levels, in a way that is analogous to a one-parameter bifurcation analysis. The difference is that the remaining parameters are allowed to vary across all of parameter space, rather than being fixed. We present our results in terms of percentages, or proportions, of parameter nodes from a given ensemble with a given property. For instance, we report the percentage of parameter nodes that have highest level of TGF *β* that admit an M state. This can be interpreted as a percentage of cell lines with the mesenchymal phenotype, with a caveat that the biologically realizable cell lines may be a strict subset of the ensemble that we consider.

As expected, E and M states dominate at the appropriate highest or lowest levels of Ovol2, Snail1, TGF *β* and Zeb1.. In fact, the E (or M) state is present in 100% of the appropriate extremal levels of Ovol2, Snail1, Zeb1 and TGF *β*, but in 45–75% of the corresponding parameter nodes this state coexists with intermediate states This multistability has consequences for the induction of EMT, since different initial parameter regimes, representing different cell lines or different genetic background, will lead to either the M or an intermediate E/M state, and likewise for MET.

Our characterization of multistability shows that the E state is exhibited in 100% of a range of parameter nodes, and likewise for M. The range depends on whether the parameter that varies is the expression of Ovol2, Snail1, Zeb1 and TGF *β*. In such a situation, even when other stable states are present, induction and then reversal of the induction will recover the original state. For instance, under TGF *β* induction, only after TGF *β* is raised to the highest level may the epithelial state transition to another state. However, in 20% of the parameter nodes at this extreme level of TGF *β*, there will be no transition out of the E state. In another 25%, the mesenchymal state is monostable (i.e. it is a single, global attractor), guaranteeing complete EMT. In the other 55% of the parameter nodes, the model indicates that the final state can be one of the intermediate states. This may explain the diversity of outcomes of EMT under induction across cell lines and across individuals.

Finally, we address the question of the number of intermediate states. In our calculations the maximal number of steady states is 8 and suggests the possibility of up to 7 experimentally observable intermediate states in some cell lines. The multistability tends to concentrate at intermediate expression levels, while monostability is almost exclusively present at the extreme values of expression levels.

### Modeling framework

We describe briefly the mathematical framework of DSGRN. The details can be found in “Methods” section and in [26, 27]. The DSGRN approach is motivated by *switching system* models, introduced by Glass and Kaufmann [32, 33], where the rate of change of each regulatory network node is governed by a piecewise constant function. The effect of node *j* onto node *i* changes from low value *L*_*ij*_ to a high value *U*_*ij*_ at a threshold *θ*_*ij*_. In addition to the three parameters 0<*L*_*ij*_<*U*_*ij*_ and 0<*θ*_*ij*_ that are associated with each edge of a network, DSGRN also considers decay rates 0<*γ*_*i*_ associated to each node of the network.

#### Parameter graph

The parameter space is a subset of $R^{3k+n}_{+}$ for a network with *n* nodes and *k* edges. The structure of the piecewise constant switching functions induces an explicit decomposition of parameter space into a finite number of regions defined by sets of inequality relationships among parameters. Each region is represented as a node in the *parameter graph*, and two nodes are adjacent if the corresponding regions share a boundary. An important feature of the parameter graph *PG* is that it is a product of *factor parameter graphs* on each node

$$PG = \Pi_{i=1}^{n} PG(i) $$

where *PG*(*i*) is the parameter graph for node *i* of the network. For more detailed and mathematically rigorous description of *PG* and *PG*(*i*), see “Methods” section and [26, 27]. An example of a factor graph is shown in Fig. 2d and is explained in more detail in “EMT model” section.

#### State transition graph

A consequence of the decomposition of parameter space is that every real-valued parameter set in $R^{3k+n}_{+}$ belongs to one of a finite number of parameter regions. Dynamics at all real valued parameters in the same region share certain important characteristics. These are captured by a *state transition graph*, which we describe in this section. The analysis of the collection of state transition graphs over all parameter nodes in the parameter graph then provides a characterization of the dynamics of a network over all of parameter space $R^{3k+n}_{+}$.

A state transition graph is a summary of trajectories that represent time evolution of gene products in the network. These trajectories evolve in phase space, which is the non-negative orthant $R^{n}_{+}$ for a network with *n* node. Because of the form of the switching system, the phase space is divided into a finite number of domains, and the directions of transitions between these domains are identical for all real-valued parameter sets in a parameter region corresponding to a single DSGRN parameter node. The state transition graph may be different at different parameter nodes. The collection of these domains can be represented as nodes of a state transition graph (STG), where two nodes may be (but are not necessarily) connected by a directed edge when the corresponding domains are adjacent i.e. share a boundary. The direction of the edge reflects the direction of the transition between the two domains. It can be shown [26] that every trajectory of the associated switching dynamical system must respect the direction of these transitions and therefore trajectories of gene expression levels are represented by paths in the STG.

More formally, the collection of thresholds divides the phase space $R^{n}_{+}$ into a finite number of *n*-dimensional cells *κ* that can be labeled by an integer vector *s*=(*s*_1_,…,*s*_*n*_), where *s*_*i*_ is the number of thresholds *θ*_*ji*_ below the *i*^*th*^ component of any point *x*∈*κ*. Let *S*_*i*_ be the range of numbers from 0 to the number of thresholds (out-edges) associated to the *i*^*th*^ node in the network. Then the set $S := \prod _{i=1}^{n} S_{i}$ can be thought of as the nodes in the state transition graph. Through a procedure using the switching system, these nodes are connected by edges that represent the dynamics of the system at the chosen parameter graph node. See “Methods” section for detailed definitions.

Figure 1 shows an example of the relationship between phase space and the nodes *S* for a two node network in which each node actuates (either represses or activates) itself and the other node. This means that there are two actuation thresholds per node, and each node can achieve states *S*_*i*_={0,1,2}. The collection of 2-dimensional cells *κ*, created by the division of $\mathbb {R}^{2}_{+}$ by the four thresholds (Fig. 1b) gives rise to the nodes of any STG for the network, which are the nine states *S*={(0,0),(1,0),(2,0),(0,1),(1,1),(2,1),(0,2),(1,2),(2,2)} in Fig. 1a.

It is useful to represent the cells *κ* as a discrete, colored grid, where the lower left and upper right corners represent the extreme values in *S*. These are (0,0) colored blue in Fig. 1c and (2,2) colored orange. Using colors along the diagonals, we represent constant Hamming distances from each of the extreme values. This is relevant when we talk about paths through phase space in “Results” section where we reference Fig. 1d.

#### Morse graph

Each state transition graph is finite, but can be quite large, and the STG grows rapidly with respect to the number of nodes and edges in the regulatory network. Therefore, to compress the information, for each STG we construct the associated *Morse graph* that retains only its set of recurrent components, which form the nodes of a Morse graph. Recall that a recurrent component in a directed graph is a maximal collection of nodes that are mutually reachable. Therefore reachability between components, when it occurs, must occur in only one direction. This reachability between the components gives rise to the Morse graph, where we assign edges between *Morse nodes* based on reachability in the STG between the components. Since reachability between components is directed, the Morse graph is acyclic.

The Morse graph summarizes the recurrent dynamics of the network. In particular, all stable steady states as well as periodic orbits will be represented as one of the nodes of the Morse graph. Stability is determined by the presence or absence of out-edges in the Morse graph. An absence of out-edges means that no other recurrent component can be reached from given recurrent component, and therefore we consider such a component *stable*. Otherwise, we consider it *unstable*.

An example Morse graph of the EMT system that we consider in this paper is given in Fig. 2c. Each node has an inscription of either FP, followed by a sequence of six numbers that represents a label in *S*, or XC. The annotation FP stands for a *fixed point* representing a steady state, and XC for a *partial cycle*; that is, a cycle where the state *s*_*i*_ is constant for at least one *i*. We append to each fixed point the state label in *S* corresponding to the location of the fixed point in phase space. In the Morse graph in Fig. 2c there are six stable steady states denoted by FP and five unstable periodic states denoted by XC. The parameter graph together with the corresponding Morse graph at each node of the parameter graph forms a *DSGRN database*.

### EMT model

We study the EMT network in Fig. 2a, taken from [11], subject to a few modifications. First, we remove the negative self-edge on Snail1, in order to define STGs unambiguously, see Remark 2 in “Methods” section. This may cause our model to miss some of the intermediate states. Second, we remove the negative regulation from Ovol2 to the edge between TGF *β* and Snail1. In our modeling paradigm, that regulation is captured by the direct negative regulation from Ovol2 to TGF *β*. Third, we separate the influences of external and internal TGF *β*. The internal TGF *β* concentration is a regular dynamic variable, whose low (high) levels may or may not activate Snail1, depending on the choice of DSGRN parameter. The influence of external TGF *β* is modeled as a shift in DSGRN parameters from (1) a DSGRN parameter where the expression of TGF *β* is never high enough to activate Snail1, through (2) a DSGRN parameter where only high level of TGF *β* expression activates Snail, to (3) a DSGRN parameter where TGF *β* is always high enough to activate Snail1.

Recall that we characterize the dynamics by a state transition graph where the level of expression is discretized using output edge thresholds. The biomarkers Ecad and Vimentin characterize the mesenchymal and epithelial states respectively, but do not have output edges.Therefore there is no natural way to subdivide their expression levels into discrete classes. We chose to characterize the E and M phenotypes without the biomarkers Ecad and Vimentin in the following way. Instead of directly tracking Ecad and Vimentin, we track the expression levels of their regulators Zeb1, Snail1 and Ovol2 (see Fig. 2a). Since Vimentin, a biomarker for the mesenchymal state, is up-regulated by Zeb1 and Snail1 and down-regulated by Ovol2, the highest expression of Vimentin will happen when Zeb1 and Snail1 are at their highest levels and Ovol2 is at its lowest level. This represents the mesenchymal state. The opposite pattern with Zeb1 and Snail1 low and Ovol2 high indicates the epithelial state where Ecad is high. Note that this is a conservative choice. It is possible, for instance, that the expression of Vimentin that characterizes the mesenchymal state does not require all three conditions (high Zeb1, Snail1 and low Ovol2); it is also possible that extreme levels of expression of these regulators are not required to induce the cell into the mesenchymal state. Making a different choice would require detailed knowledge of the numerical values of parameters that we do not have. If such information becomes available, it would restrict the set of parameter nodes that we consider to a smaller set of those that would be consistent with such data.

We assign the highest and lowest levels of expression in terms of the state labels in *S*. After the removal of Ecad and Vimentin, Zeb1 has three output edges in the network, and hence Zeb1 can attain four states 0,1,2,3. Snail1 also has three output edges after the additional removal of the negative self-regulation, so it also can attain four states 0,1,2,3. Finally, Ovol2 has two output edges and so it can attain states 0,1,2. By choosing the order of the states of the genes to be

$$\qquad \text{ (Zeb1, Snail1, miR200, miR34a, TGF\(\beta\), Ovol2)},$$

we represent the mesenchymal state by an FP state of the form

$$M= \text{FP}(3,3, *,*,*,0),$$

and the epithelial state by an FP state of the form

$$E= \text{FP}(0,0,*,*,*,2),$$

where the symbol ∗ allows any state of the other genes. The regulator miR200 has a highest state of 2, miR34a has a highest state of 1, and TGF *β* has a highest state of 1.

Notice that the epithelial state is present in the Morse graph in Fig. 2c in the lower left. The Morse graph shows multistability between E together with five intermediate E/M states. For example, FP(2,0,1,1,0,1) represents a FP steady state where Snail1 and TGF *β* are at their lowest level, miR200, Ovol2, and Zeb1 are at intermediate levels, and miR34a is at its highest level. In “Results” section we will discuss our findings regarding intermediate E/M states in detail.

Since the EMT network in Fig. 2b has 6 nodes and 12 edges, parameter space is 6+3∗12=42 dimensional. The corresponding parameter graph has more than 21 billion parameter nodes, each associated to a region in 42-dimensional parameter space. If we want to query the parameter graph for changes in steady states induced by changing expression level of a particular gene, like TGF *β*, we will use the factor parameter graph *PG*(*i*) for the gene *i* to represent these changes (see “Modeling framework” section.)

As an illustration, we describe an example of *PG*(*k*) where node *k* has one input edge and two output edges, as is true for Ovol2 in the EMT network. This factor graph is shown in Fig. 2d. Ovol2 has a single in-edge from Zeb1 and two out-edges to Zeb1 and TGF *β*. For simplicity, denote *γ*_*Ovol*2_, the degradation rate of Ovol2, by *γ*, and denote *L*_*Ovol*2,*Zeb*1_ and *U*_*Ovol*2,*Zeb*1_ by *L* and *U*, respectively. Recall that parameter nodes are associated to regions in parameter space defined by inequalities (see “Parameter graph” section for more detail). The inequalities corresponding to each of the parameter nodes in the factor parameter graph for Ovol2 are:

1 $$ \begin{aligned} &A1: (L <U <\gamma \cdot \theta_{Zeb1,Ovol2}<\gamma \cdot\theta_{TGF\beta,Ovol2})\\ &A2: (L<\gamma \cdot \theta_{Zeb1,Ovol2}<U<\gamma\cdot\theta_{TGF\beta,Ovol2})\\ &A3: (\gamma\cdot\theta_{Zeb1,Ovol2}<L<U<\gamma\cdot\theta_{TGF\beta,Ovol2})\\ &A4: (L<\gamma\cdot\theta_{Zeb1,Ovol2}<\gamma\cdot\theta_{TGF\beta,Ovol2}<U)\\ &A5: (\gamma\cdot\theta_{Zeb1,Ovol2}<L<\gamma\cdot\theta_{TGF\beta,Ovol2}<U)\\ &A6: (\gamma\cdot\theta_{Zeb1,Ovol2}<\gamma\cdot\theta_{TGF\beta,Ovol2}<L<U)\\ &B1: (L <U <\gamma\cdot\theta_{TGF\beta,Ovol2}<\gamma\cdot\theta_{Zeb1,Ovol2})\\ &B2: (L<\gamma\cdot\theta_{TGF\beta,Ovol2}<U<\gamma\cdot\theta_{Zeb1,Ovol2})\\ &B3: (\gamma\cdot\theta_{TGF\beta,Ovol2}<L<U<\gamma\cdot\theta_{Zeb1,Ovol2})\\ &B4: (L<\gamma\cdot\theta_{TGF\beta,Ovol2}<\gamma\cdot\theta_{Zeb1,Ovol2}<U)\\ &B5: (\gamma\cdot\theta_{TGF\beta,Ovol2}<L<\gamma\cdot\theta_{Zeb1,Ovol2}<U)\\ &B6: (\gamma\cdot\theta_{TGF\beta,Ovol2}<\gamma\cdot\theta_{Zeb1,Ovol2}<L<U) \end{aligned}  $$

Note that the difference between the *A* and *B* nodes is simply the ordering of the two thresholds.

Importantly, some of these inequalities represent parameter choices when the network does not work as depicted in Fig. 2a. For instance, node A1 implies that the output edges from node Ovol2 will never get actuated for any choice of inputs. On one hand this does represent a very low level of expression of gene Ovol2 which is a valid state of this gene. On the other hand, at this parameter node the output edges from node Ovol2 do not carry any information. Therefore removing these edges will produce the same dynamics. In other words, the dynamics of the network at this parameter node are equivalent to the dynamics of a subnetwork. We say that this node is an *inessential* parameter node. Nodes that are not inessential are essential. In the above example, the nodes A4 and B4 are essential, while the other nodes are inessential. Therefore A4 and B4 comprise the *essential factor parameter graph* for Ovol2.

An *essential parameter graph* is a product of essential factor parameter graphs. This is usually much smaller than the entire parameter graph, since the latter describes not only dynamics of the network, but also dynamics of all its subnetworks. In the EMT network, the full parameter graph, which includes both essential and inessential parameter nodes, represents over 21 billion parameter regions. The essential parameter graph has only about 21 million parameter regions, a thousand-fold reduction in size. For overall statistics of the EMT network, we will use the essential parameter graph. When tracking the changing abundance of a gene product, we will compute the parameter graph with essential and inessential parameter nodes for that gene, and only essential parameter nodes for all other genes.

## Results

One of the key questions in the EMT process is understanding the diversity of the intermediate steady states between the epithelial and mesenchymal phenotypes and how these states are activated and deactivated during the EMT and MET transitions [17]. These states may represent partial phenotypes that could be experimentally characterized and then perhaps pharmacologically controlled. Previous modeling work using differential equations models considered one or two parameters at a time and found up to two intermediate steady states. In the following analysis, we characterize the number and location of intermediate E/M states as found by DSGRN using the network in Fig. 2b. Our method is somewhat analogous to a one-parameter bifurcation analysis, but the difference is that the remaining parameters are allowed to vary across all of parameter space, rather than being fixed.

We choose to concentrate on four key variables: TGF *β*, Ovol2, Snail1, and Zeb1. TGF *β* is a well-known inducer of EMT [12, 17, 19] and recent work has shown that over-expression of Ovol2 restricts EMT and drives MET [11, 34]. In [34] the authors performed bifurcation analysis to explore the response of the miR200/Zeb1/Ovol2 circuit to different levels of Snail1. They have shown that as Snail1 increases EMT is induced. Furthermore, during MET, when Snail1 levels are decreased, mesenchymal cells initially undergo a partial MET to attain an intermediate E/M phenotype and after a further decrease in Snail1, MET is completed.

We compute four DSRGN databases. In the first, we allow the Ovol2 factor parameter graph to include both essential and inessential nodes, while all the other factor parameter graphs corresponding to the other genes are essential. We call this the *Ovol2-general parameter graph.* We then compute the Snail1-, TGF *β*- and Zeb1-general parameter graphs as well. Clearly, the intersection between all four of these parameter graphs is the essential parameter graph.

The Ovol2 parameter factor graph is shown in Fig. 2d, and the extreme points *A*1 and *B*1 correspond to Ovol2 at its lowest level. In other words, *A*1 and *B*1 represent parameter regions in which Ovol2 is always below all thresholds at which it actuates its downstream genes. Likewise, the points *A*6 and *B*6 represent parameter regions where Ovol2 is at its highest level, and above all thresholds for the actuation of downstream targets. The parameter nodes in between these extremes represent a gradual increase in Ovol2 expression levels as measured by the number of downstream genes it actuates. To facilitate graphing dynamical properties as functions of increasing abundance of Ovol2, we compress the structure of the factor graph (Fig. 2d) into five *layers* denoted by the numbers on the horizontal axis representing qualitative Ovol2 expression levels. As the layer number increases by one, the Ovol2 expression level is able to actuate more of its downstream genes. The layers of the factor parameter graphs for other genes are also compressed in this way. The complexity of the factor parameter graph and thus the number of its layers depends on the number of inputs and outputs of the node; more complex nodes have more complex factor parameter graphs. We will report prevalence of different dynamical features for each layer.

We tabulate where different types of FPs occur in parameter space. For every parameter in the Ovol2-general parameter graph, the projection of that parameter onto the Ovol2 factor graph in Fig. 2d occurs in one of the five layers. For each layer in the Ovol2-general parameter, we count how many times a given type of FP occurs. That is a measure of the prevalence of that FP within the parameter graph as a function of increasing Ovol2.

In addition to the location in a layer of the Ovol2 factor parameter graph, every FP has a location in phase space. The location in phase space is encoded in the 6-dimensional vector of integers that places the FP in the discrete grid given by the thresholds of the system. See Fig. 1a-c for a 2D example, and Fig. 1d for the discrete grid for the EMT network in TGF *β*, Ovol2, and Snail1, and see “Methods” section for more mathematical detail.

Because the expression of the mesenchymal marker Vimentin and the epithelial marker Ecad are fully determined by the expression of Ovol2, Snail1 and Zeb1, the degree to which an FP state is mesenchymal vs. epithelial is determined by the projection of the 6-dimensional vector onto these three variables. In this projection (Fig. 1d) the extreme points on the opposing ends of a diagonal represent the E state (0,0,∗,∗,∗,2) (dark blue) and M state (3,3,∗,∗,∗,0) (orange). Furthermore, the Hamming distances from these extremes characterize the degree to which any of the intermediate states resemble mesenchymal vs. epithelial states. We depict the Hamming distance one diagonal away from the epithelial state in light blue, distance two in violet, distance 3 in light orange, etc. We will report the number of FPs in each phase space diagonal to show the distribution of various types of intermediate steady states across this projection of phase space.

Our first result justifies our restricted definition of the epithelial state given in “Modeling framework” section. In all our queries for all essential nodes in Snail1-general, TGF *β*-general, and Zeb1-general cases, any state of the form (3,3,*,*,*,*) is actually a state with last component (Ovol2) equal to zero (3,3,*,*,*,0). In addition, every state of the form (0,0,*,*,*,*) is actually of the form (0,0,*,*,*,2). So in these cases by requiring that in the epithelial state Ovol2 expression is high we did not lose any epithelial states.

Our second set of results concerns the types of attractors that the EMT network can exhibit, the frequency with which we observe the E and M states, and how often the E and M states are *monostable*. An attractor is monostable if it is the only stable node in the Morse graph. *Multistability* of attracting states means that multiple stable Morse nodes are present in the Morse graph (see e.g. Fig. 2c). We observe that in all parameter nodes there are only fixed point attractors. As illustrated in Fig. 2c there are Morse nodes with signature XC, which correspond to closed state transition paths along which several gene product abundances oscillate. However, these are always unstable in the model and so likely not experimentally observable, or observable only as transients. Therefore the EMT network structure robustly exhibits stable steady states FP despite the complicated feedback interactions, and oscillations play a role only as parts of the boundary between basins of attraction of different FPs.

Interestingly, all 21 million nodes in the essential parameter graph exhibit only multistability and never monostability. Furthermore, every one of the essential parameters has both E and M states as stable steady states, indicating that the epithelial and mesenchymal states are highly prevalent across parameter space. When we start examining inessential parameter nodes, we do see monostability, although most parameters still exhibit multistability. The appearance of monostability only at the inessential nodes indicates that our EMT network model is subject to control via low or high levels of particular gene products, consistent with experimental results [11, 12, 20, 34].

**Fig. 3:**
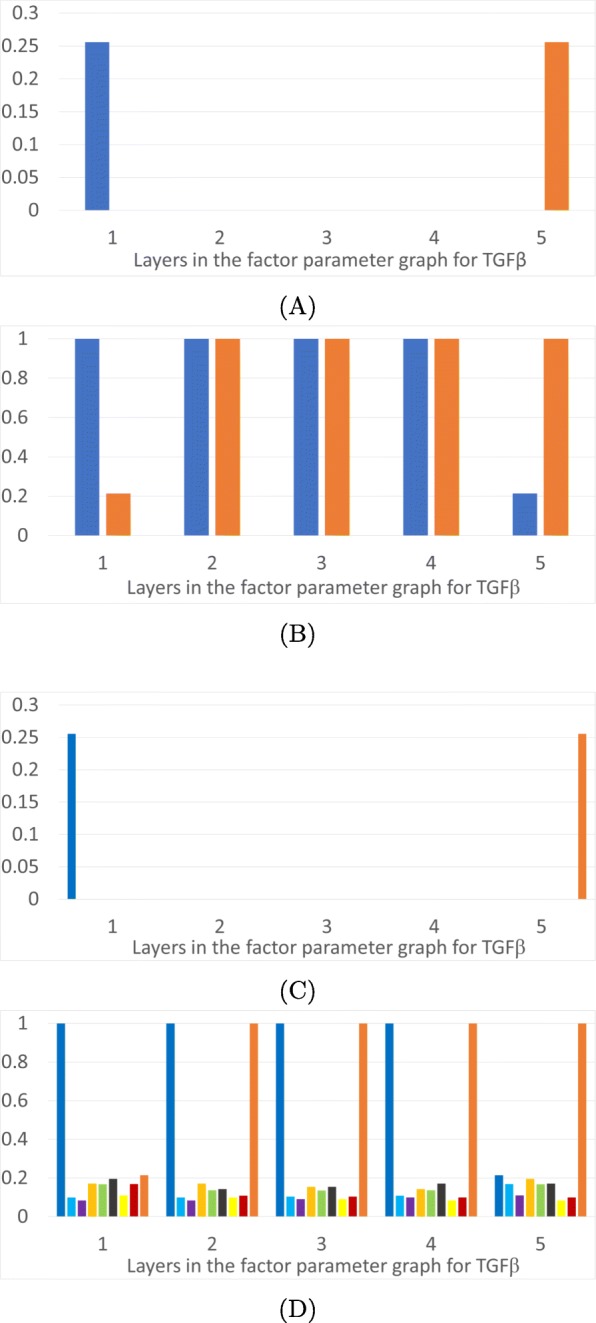
Epithelial and mesenchymal states as a function of level of TGF *β*. The horizontal axis is the five layers in the factor parameter graph for TGF *β*, which is isomorphic to half of the factor parameter graph for Ovol2 in Fig. 2d. **a**: Proportions of parameter nodes with monostable E (dark blue) or M (orange) states. **b**: Proportions of parameter nodes with the occurrence of E or M in each layer of the TGF *β* factor parameter graph. **c**: Proportions of parameter nodes with monostable FP in color coded layers of the 3D projection of the phase space in Fig. 1d. **d**: Proportions of parameter nodes that exhibit an FP, not necessarily monostable, in color coded layers of the 3D projection of the phase space in Fig. 1d

**Fig. 4:**
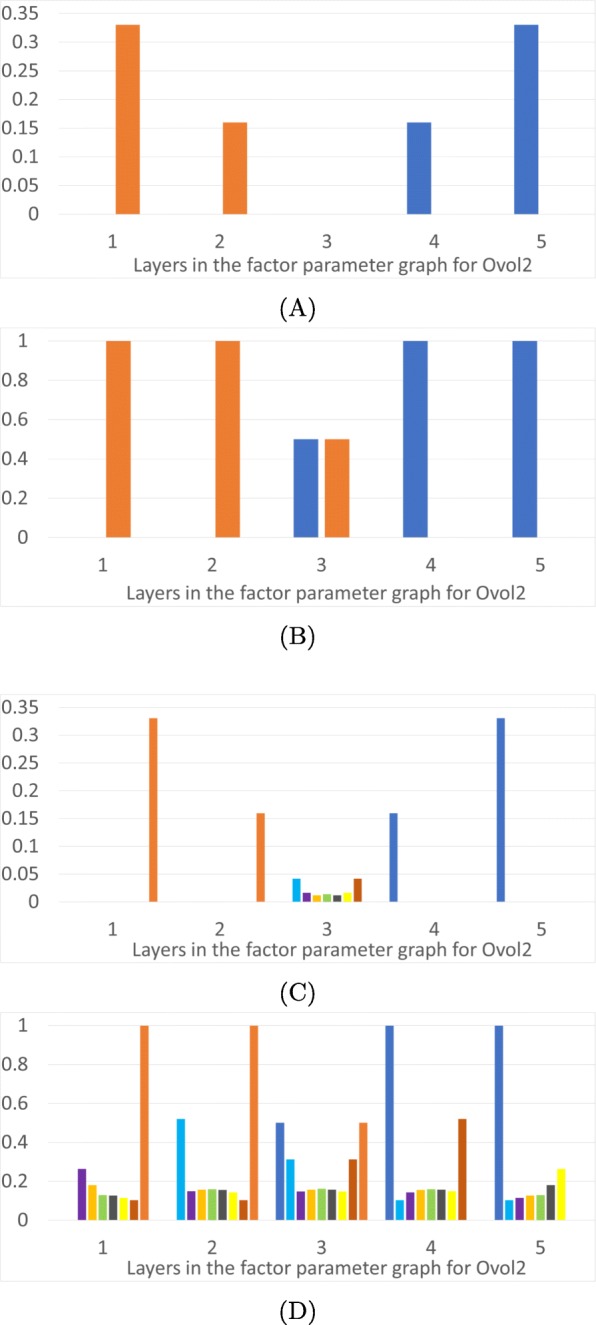
Epithelial and mesenchymal states as a function of level of Ovol2. The horizontal axis is the five layers in the factor parameter graph for Ovol2, see Fig. 2d. **a**: Proportions of parameter nodes with *monostable* E (dark blue) or M (orange) states. **b**: Proportions of parameter nodes with the occurrence of E or M in each layer of the Ovol2 factor parameter graph. **c**: Proportions of parameter nodes with monostable FP in color coded layers of the 3D projection of the phase space in Fig. 1d. **d**: Proportions of parameter nodes that exhibit an FP, not necessarily monostable, in color coded layers of the 3D projection of the phase space in Fig. 1d

**Fig. 5:**
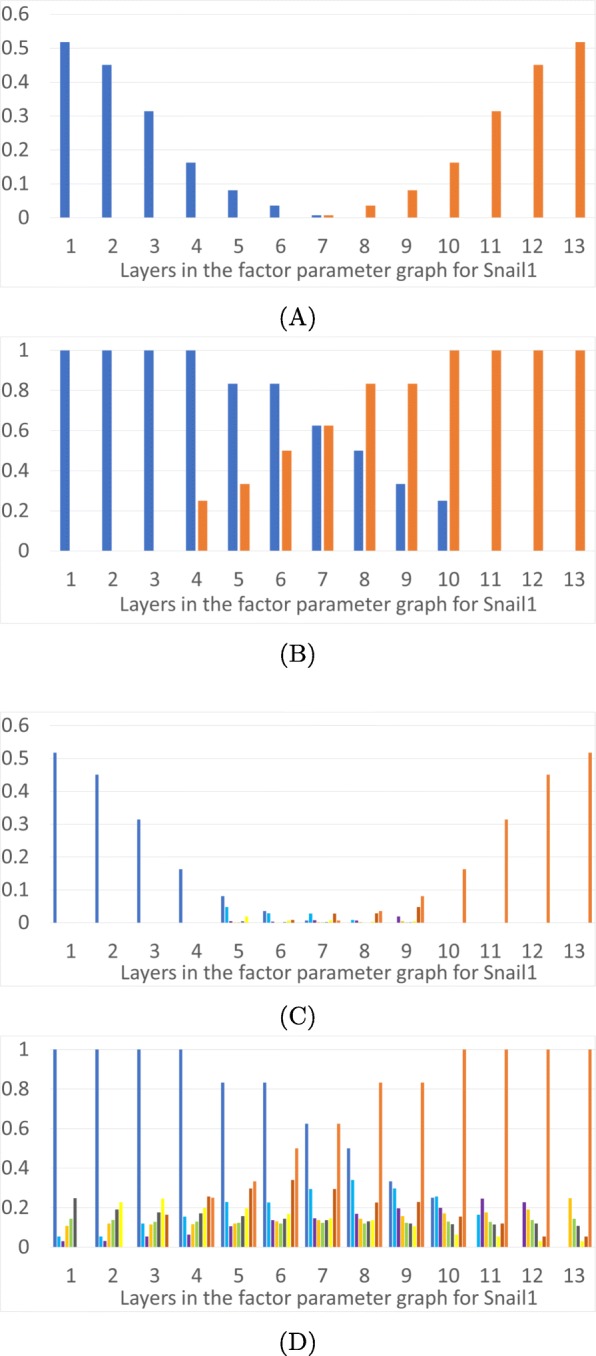
Epithelial and mesenchymal states as a function of level of Snail1. The horizontal axis is the 13 layers in the factor parameter graph for Snail1. **a**: Proportions of parameter nodes with monostable E (dark blue) or M (orange) states in each layer of the factor parameter graph on Snail1. **b**: Proportions of parameter nodes with the occurrence of E or M in each layer of the Snail1 factor parameter graph. **c**: Proportions of parameter nodes with monostable FP in color coded layers of the 3D projection of the phase space in Fig. 1d. **d**: Proportions of parameter nodes that exhibit an FP, not necessarily monostable, in color coded layers of the 3D projection of the phase space in Fig. 1d

**Fig. 6:**
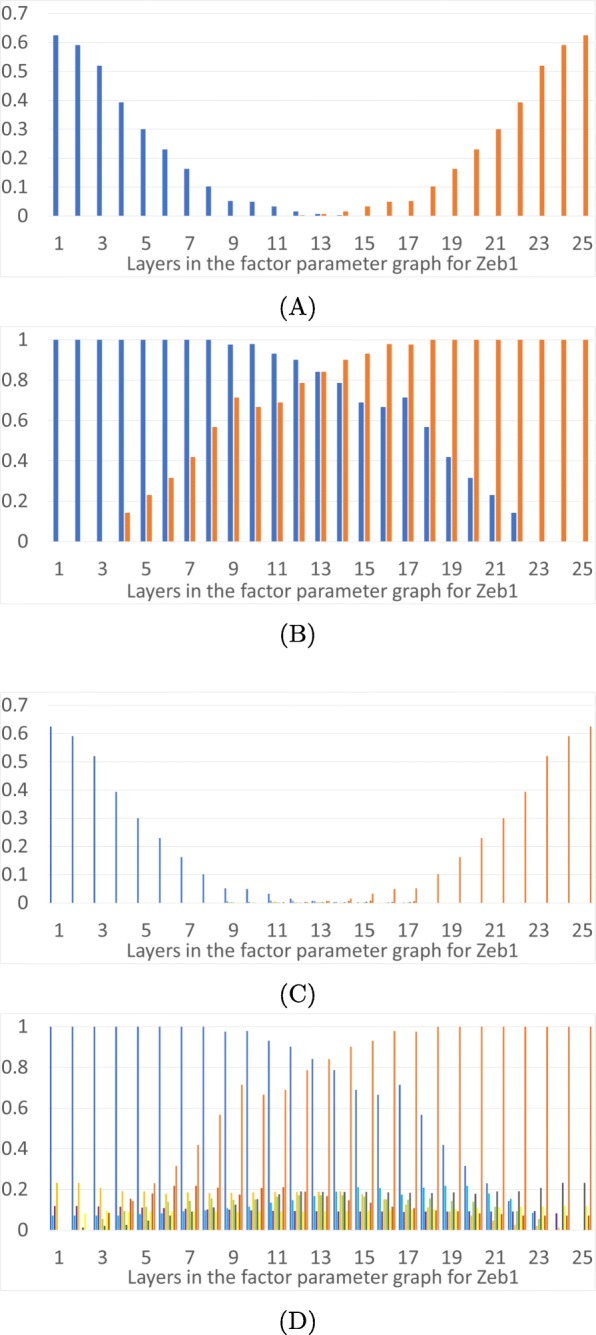
Epithelial and mesenchymal states as a function of level of Zeb1. The horizontal axis is the 25 layers in the factor parameter graph for Zeb1. **a**: Proportions of parameter nodes with monostable E (dark blue) or M (orange) states in each layer of the factor parameter graph on Zeb1. **b**: Proportions of parameter nodes with the occurrence of E or M in each layer of the Zeb1 factor parameter graph. **c**: Proportions of parameter nodes with monostable FP in color coded layers of the 3D projection of the phase space in Fig. 1d. **d**: Proportions of parameter nodes that exhibit an FP, not necessarily monostable, in color coded layers of the 3D projection of the phase space in Fig. 1d

We now describe monostability vs multistability of FPs over the phase space as a function of parameters. In Fig. 3 we present results for the TGF *β*-general parameter graph, in Fig. 4 results for the Ovol2-general parameter factor graph, in Fig. 5 results for Snail1-general parameter graph, and in Fig. 6 results for Zeb1-general parameter graph. In each figure we present a frequency of particular FP states (vertical axis) as a function of layers of the factor parameter graph.

The factor graphs for TGF *β*, Snail1 and Zeb1 are different than the one for Ovol2 in Fig. 2d. TGF *β* has two in-edges and one out-edge, Snail1 has two in-edges and three out-edges, and Zeb1 has three in-edges and three out-edges, as shown in Fig. 2b, unlike the one in-edge, two out-edge topology of Ovol2. The factor graph of TGF *β* is isomorphic to the A1-A6 half of the Ovol2 factor graph in Fig. 2d, and so has five layers like Ovol2. Snail1 has a far more complex factor graph with 300 nodes and 13 layers. Zeb1 factor graph has 4242 nodes in 25 layers.

Figures 3, 4,5 and 6 show the overall distributions of the E, M and intermediate E/M states. Before we go into the details, we point out that the results we will discuss shortly are in broad agreement with previous theoretical and experimental results [11, 12, 19, 21, 34]. In particular, over-expression of Ovol2 restricts EMT and drives MET, knockdown of Ovol2 may lead to EMT, an increase in the expression level of TGF *β* may drive EMT, and an increase (decrease) in the expression level of Snail1 and Zeb1 can potentially drive EMT (MET).

In part (A) of each figure we present the frequencies of *monostable* E and M states. At those parameters exhibiting monostability, no other phenotypic state is achievable. These states are more prevalent at the extremes of the parameter space: the monostable E state occupies 25% of low levels of TGF *β* (Fig. 3a) and 33% of the high expression levels of Ovol2 (Fig. 4a). Interestingly, for TGF *β* all the monostable E states are at the lowest value, while Ovol2 experiences a sharp drop-off in number of monostable E states at the third layer. The situation is more interesting for Snail1 and Zeb1. The E state dominates at low levels of Snail1 but the frequency of the monostable E state only gradually decreases as Snail1 levels increase. We remark that this may partially be an artifact of the larger number of factor graph layers in Snail1 and Zeb1.. However, it is also notable that >50% parameters exhibit monostable E at the lowest levels of Snail1. Therefore, the monostable E state does seem to be substantially more prevalent in the Snail1-general parameter graph. Situation is similar for Zeb1. The E state dominates at low levels of Zeb1 where 62*%* parameters exhibit monostable E at the lowest levels of Zeb1. The frequency of the monostable E state only gradually decreases as Zeb1 levels increase. The M state dominates at the opposite values of these three variables, with the identical frequencies.

In each Figure panel (B) extends the analysis in panel (A) by including not only monostable E and M states, but all E and M states that occur in the system. The difference between panels (A) and (B) describes those E and M states that are parts of multistable configurations of steady states FP. In all three projections the middle layers include a significant proportion of states E and M participating in multistable configurations.

It is remarkable that both E and M states are present in all parameter nodes in the middle three layers of the TGF *β* projection in Fig. 3b. This indicates that if a system starts in the epithelial state at low expression of TGF *β* (layer 1) and then TGF *β* is raised to second to highest value (layer 4), the system will stay in the epithelial state. Even more remarkably, if TGF *β* is raised to its highest value (layer 5) there are still 20% of parameter nodes where the E state exists. This can be interpreted to mean that 20% of the cell lines do not convert to the mesenchymal state even under very high TGF *β* levels, unless there is a secondary external perturbation not modeled by this network. Furthermore, out of the remaining 80% of the parameter nodes, only 25% are in the monostable mesenchymal state, which guarantees the completion of EMT. In the remaining 55% of the parameter nodes, the model indicates that even under a high level of TGF *β* some cells lines may not complete the full transition to the mesenchymal state. This may explain the diversity of outcomes of EMT under induction across cells lines and across individuals.

An increase in Snail1 also induces EMT, but the epithelial state does not continue across all layers. Increasing Snail1 to layer 11 will perturb the system away from the epithelial state. However, since the mesenchymal state is monostable only in about 30% of layer 11, 70% of the parameter nodes have the potential to go to one of the intermediate E/M states upon leaving the epithelial state. Even at the highest values of Snail1, just above 50*%* of parameter nodes lead to the monostable M state; for other parameters, the system may not be in the M state at a very high level of Snail1. Similarly, an increase in Zeb1 induces EMT and increasing Zeb1 to layer 23 will perturb the system away from the epithelial state; at the highest levels of Zeb1, at 48% the system may not be in M state.

Similarly, induction of MET by increasing concentration of Ovol2 is guaranteed to transition out of the mesenchymal state, since there is no mesenchymal state in layers 4 and 5 in Fig. 4b. The state the system transitions to is guaranteed to be the epithelial state in 33% of parameter nodes, since 33% of parameter nodes are monostable E states in Fig. 4a. In other cases the final state of the MET induction can be one of the intermediate states, represented in layer 5 of Fig. 4d, most of which are in domains that are close (in Hamming distance) to E. This is compatible with the results of Hong [11], who experimentally observed that the mesenchymal state is “lost” before the epithelial state is reached under Ovol2 expression.

To understand the distribution of intermediate FPs in the three dimensional projection depicted in Fig. 1d we present panels (C) and (D) in Figs. 3, 4, 5 and 6. The colored frequency bars in panels (C) and (D) refer to the number of parameters with an FP that lies within the associated diagonal in Fig. 1d. In panel (C) we show proportions of parameters with monostable FPs including the E/M intermediate states in each layer, and in panel (D) all FPs in these layers, including E/M intermediate phenotypes in multistable configurations. While the monostable intermediate states concentrate in the middle layers, what is remarkable is that in a significant percentage of parameter nodes there are intermediate states at the extreme values of all three gene products. This is especially significant in Ovol2. This shows that based on the parameters of the system, the induction of an epithelial state may not end in a mesenchymal state, but in one of the many intermediate states. Note that in all extremal parameter regimes each intermediate state is in a multistable regime where one of the other steady states is either an M state or an E state, since these occur in 100% of the extremal parameters. These observations may explain the diversity of outcomes of EMT under induction across cells and across individuals. Moreover, the wide distribution of the intermediate states in various phase space diagonals and the gradual disappearance of the parameter nodes with E or M state in extremal layers confirm the possibility that EMT and MET produce cells residing within a spectrum of intermediate phenotypic states, where cells advance to differing extents through these programs, progressively acquiring the new phenotypic features as they shed the features of their original state, as stated in [17].

**Fig. 7:**
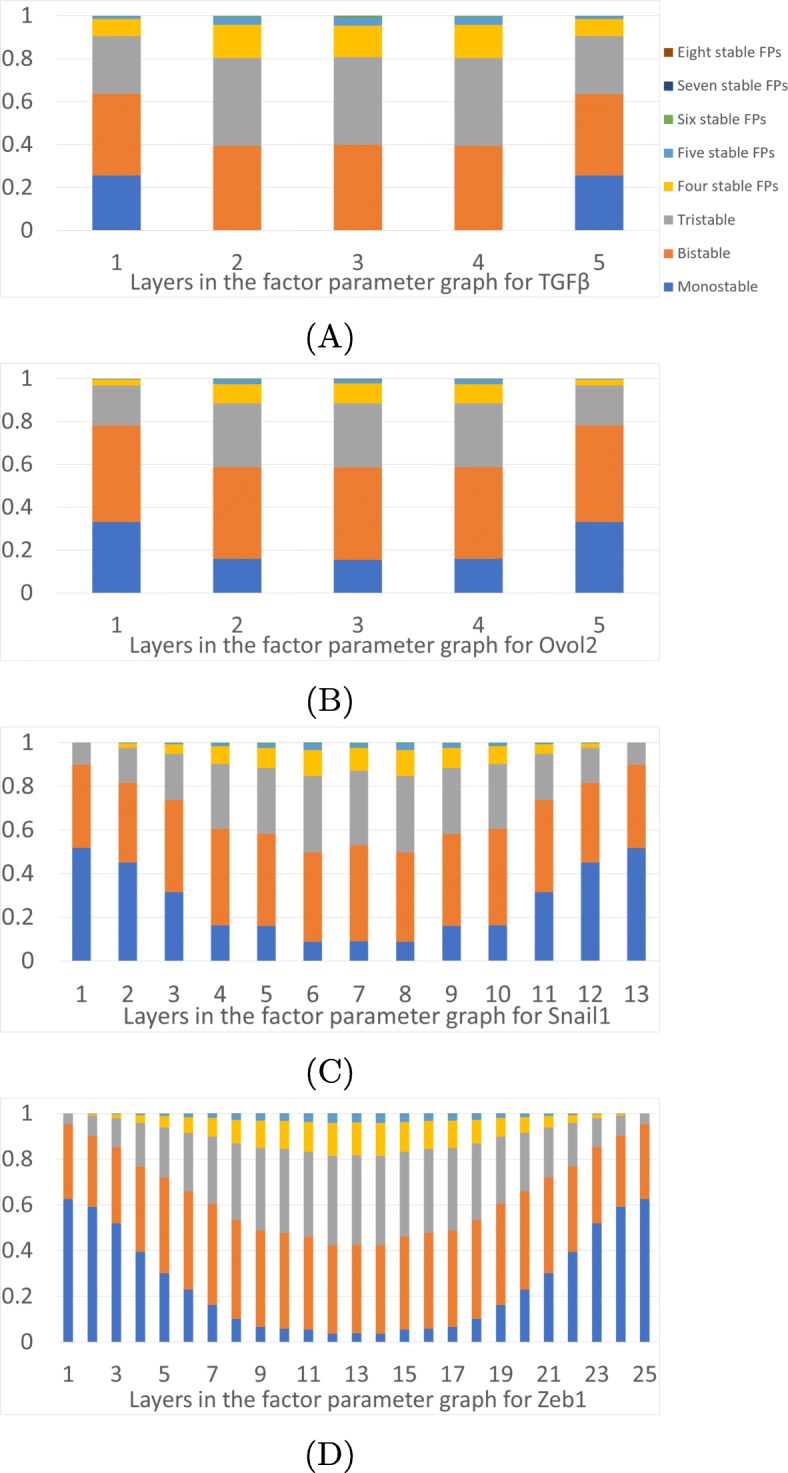
Prevalence of multistability. Proportions of the parameter nodes in each layer of the factor parameter graph that exhibit *k*-stability, *k*=1,…,8. **a** TGF *β*, **b** Ovol2, **c** Snail1, and **d** Zeb1

We offer a general picture of the multistability in the EMT network by presenting a summary of the number of fixed points FP in a Morse graph as a function of the layers in the factor parameter graphs. In Fig. 7 we show proportions of parameters in different layers of the parameter graphs of TGF *β*, Ovol2, Snail1 and Zeb1 that exhibit *k*-multistability (i.e. *k* stable fixed points). Two main observations are that the multistability is not evenly distributed in the parameter graph. The extreme values of parameters are dominated by monostability and low *k* multistability. For Ovol2 extreme values there are at most five stable FP steady states, for Snail1 and Zeb1 extreme values there are at most three stable FP states, but for TGF *β* there is also 6-multistability. The proportions of the parameters for some of the higher-multistability cases can be too small to be visually distinguished in Fig. 7. All the highest *k* multistability is concentrated in the central regions of the parameter graph. For essential parameter nodes, which lie in the intersection of all three presented data sets, the maximal number of coexisting stable FP steady states is seven. Since these always include both E and M states, for the essential nodes there are at most five intermediate FP states (intermediate phenotypes). However, if we allow the parameter nodes for Ovol2 or TGF *β* to be inessential, we find eight coexisting FP steady states, where for the Ovol2-general parameter graph, only one of the E and M states has to occur among the eight stable coexisting FP states. Hence there are seven intermediate stable steady states FP in Ovol2-general graph that can coexist. In the TGF *β*-general parameter graph, both E and M states are always among the eight, hence there are at most six intermediate stable FP states.

Finally, we asked if an FP can occupy any state in six dimensional phase space. That is, given any possible collection of six integers denoting the level of each gene product, is there some parameter where that state is a fixed point? There are 576 possible domains in the phase space and therefore 576 possible six dimensional FP annotations. Out of these 576 domains, the FPs generated by Ovol2-general parameter graph occur in 238 (41.3*%*), FPs generated by the Snail1-general parameter graph occur in 162 (28.1*%*) and FPs generated by TGF *β*-general parameter graph occur in 124 (21.1*%*). Therefore only a minority of the domains admit an FP.

## Discussion

Mathematical models based on ODEs face significant challenges when modeling complex networks. The selection of nonlinearities is not based on first principles, parameters are largely unknown, and initial conditions are mostly not measurable. Given that the simplified EMT network has six dimensional phase space and dozens of parameters, making inferences about the model dynamics from network structure by sampling parameters and sampling initial data leads to clear challenges of interpretability and generality of the results.

In this paper we present an alternative analysis of the complex EMT network, which is based on a different approach to dynamical systems. In dynamical systems, the first major transition from an emphasis on finding individual solutions to seeking understanding of invariant sets and long-term dynamics took place more than 100 years ago and was initiated by Poincaré. However, in the 100 years since then we have learned that the invariant sets do not behave robustly with respect to parameters [35, 36]. To address the lack of robustness of invariant sets with respect to parameters, another change in perspective is needed. Initiated by C. Conley [37] and developed over the last 40 years [38–41] the emphasis shifts from invariant sets to *positively attracting sets*.

This theory has found a computable implementation in DSGRN (Dynamic Signatures of Gene Regulatory Networks) [26, 28–31] which enables computation of lattices of attracting sets and Morse graphs across all parameters for a given regulatory network. This approach has been applied [27] to the E2F-Rb signaling network that controls the G1/S transition in mammalian cell cycle. We are not aware of any other approach, apart from sampling parameter space [24] and simulation, to understand how a complex system behaves with respect to (dozens of) parameters.

This approach allows a global view of the dynamics. We investigate monostability and multistability and find that monostability dominates at the low and high expression levels of Ovol2, Snail1 and TGF *β*. In the middle values we see the presence of *k*-multistability with *k*≤8. Multistability with smaller *k* is present even at the extreme values of the expression levels. This can be interpreted as an indication that the effect of the induction of the EMT (or MET) may not be the target E or M state, but some of the intermediate states.

In our approach, the phase space is divided into a fixed number of domains based on thresholds of activation/deactivation of different genes. Attractors in the state transition graph that consist of a single domain are interpreted as stable states of the system and are assigned a signature that identifies the domain. Therefore by design there are only a finite number of types of steady states that the system may have. We identify two such signatures FP(3,3,*,*,*,0) and FP(0,0,*,*,*,2) as mesenchymal and an epithelial states, respectively, since they represent the appropriate mixture of highest and lowest expression levels of Zeb1, Snail1 and Ovol2. This rigidity has the advantage of a clear definition of what E and M states are; however, it is not immediately clear if this is a valid biomedical interpretation. For instance, it may be that the states having slightly smaller expression levels of either Snail1 or Zeb1 FP(3,2,*,*,*,0) and FP(2,3, *,*,*,0) also represent epithelial states. The same comment applies to intermediate states. The fact that we found parameter nodes with six intermediate stable states in addition to E and M states is an indication of richness of the EMT dynamics, but it is not clear if there indeed are six clinically distinct intermediate E/M states.

Each DSGRN state transition graph can be related to the limit of a Hill function model at a particular set of parameters as the Hill coefficient grows without bound. Each DSGRN parameter node describes a set of inequalities which generate a particular state transition graph. Therefore each parameter node that exhibits dynamics of interest can be translated to a set of Hill function models whose parameters satisfy the same inequalities and differ only by a choice of the Hill coefficient.

This correspondence can be used to focus attention on particular parts of the parameter graph. For instance [20] has shown the central role of the bistable modules miR200-Zeb1 and miR34a-Snail1 in the EMT transition. They found that the first is tristable, while the second is monostable. In the DSGRN approach, this corresponds to a subset of parameter nodes in parameter factor graphs for miR200, Zeb1, miR34a, and Snail1. With these parameter nodes fixed, one can then investigate how a choice of parameter node in parameter factor graphs for Ovol2, TGF *β* affects the number and type of steady states, as well as the sequencing of transitions between E and M states [19].

Our approach opens up possibilities for studying important questions about how multistability in the EMT network affects the diversity of outcomes after induction. Multistability is always accompanied by hysteresis and thus potential lack of reversibility of the partial induction. Furthermore, the difference in network parameters at the start of the induction may result in a different sequence of intermediate states during the process of induction as well as a different final state. The same is true for partial inductions; the initial network parameters will determine how much of partial induction is fully reversible, and what the final state is.

## Conclusions

We present an alternative analysis of the complex EMT network, which is based on an approach that allows a coarse representation of the dynamics across the entire range of parameters. This global view of the dynamics indicates that multistability is highly prevalent in the EMT network. Multistability, when accompanied by a complex web of hysteresis relationships, can lead to a greatly variable final state of the system under variable sequences of increases and decreases of induction signals. This suggests that the cellular state subject to a partial induction of EMT transition, or repeated increase and decrease of the induction signals, may transition to states which may sensitively depend on the initial state, amount, and duration of the periods of increases and decreases of induction signals. These states, in turn, may lead to highly variable clinical outcomes.

## Methods

In this section we describe our mathematical model and the basic concepts of DSGRN, which allows a finite description of the network dynamics across phase space and parameter space. The details can be found in [26, 27].

A regulatory network **RN** is a finite directed graph with edges annotated by *j*→*i* or *j*⊣*i*, representing node *i* activated or repressed by node *j*, respectively. There is at most one edge from one node to another. Let *n* be the number of nodes in a regulatory network throughout this section.

### Switching system

In this work we use a particular differential equation model for network dynamics, called a *switching system*, introduced by Glass and Kaufmann [32, 33]:

2 $$  \dot{x}_{i} = - \gamma_{i} x_{i} + \Lambda_{i}(\sigma_{ij_{1}}^{\pm}(x_{j_{1}}), \ldots, \sigma_{ij_{q}}^{\pm}(x_{j_{q}})), \qquad i = 1, \ldots,n,  $$

where *x*_*i*_ represents the concentration of node *i*, *γ*_*i*_ denotes the rate of degradation of *x*_*i*_, and each instantiation of *σ*^±^ is either *σ*^+^ or *σ*^−^, representing either up- or down- regulation of *x*_*i*_ by $x_{j_{k}}$, respectively. The number *q*=*q*(*i*) is the number of input edges in **RN** to node *i*.

To each edge *j*→*i* or *j*⊣*i* in a regulatory network, DSGRN assigns three parameters *L*_*ij*_,*U*_*ij*_ and *θ*_*ij*_, with 0<*L*_*ij*_<*U*_*ij*_ representing low and high levels of growth of *x*_*i*_ that are determined by the value of *x*_*j*_ relative to the threshold *θ*_*ij*_>0. The collection of decay parameters *γ*_*i*_, *i*=1,…,*n*, and triples (*U*_*ij*_,*L*_*ij*_,*θ*_*ij*_), one for each edge *j*→*i* or *j*⊣*i*, forms a parameter space for the network. The piecewise constant functions *σ*^±^ are written

$$\sigma_{ij}^{+}(x_{j}) =\left \{ \begin{array}{cc} U_{ij} & \text{ if} x_{j} > \theta_{ij} \\ L_{ij} & \text{ if} x_{j} < \theta_{ij} \end{array} \right., \quad \sigma_{ij}^{-}(x_{j}) =\left \{ \begin{array}{cc} L_{ij} & \text{ if} x_{j} > \theta_{ij} \\ U_{ij} & \text{ if} x_{j} < \theta_{ij}. \end{array} \right. $$

The function *Λ*_*i*_ in (2) is a multi-linear function describing how the values $\sigma ^{\pm }_{ij}$ are combined. Based on biological considerations we assume that $\Lambda _{i} = \prod \sum \sigma _{ij}^{\pm }(x_{j})$ is a product of sums, where each subscript (*ij*) occurs at most once (see [26] for more detail). The collection of functions *Λ*_*i*_, *i*=1,…,*n* must be specified along with the structure of the network. For a node *i* with *q* incoming edges, the domain of *Λ*_*i*_ is a set of 2^*q*(*i*)^*input sequences*:

$$A_{i} := \{(\alpha_{ij_{1}},\ldots, \alpha_{ij_{k}}): \alpha_{ij_{k}} \in \{L_{ij_{k}}, U_{ij_{k}}\}, 1 \leq k \leq q(i)\}.$$

The switching system that we use to model EMT is

$$\begin{array}{*{20}l} \dot{g} &= -\gamma_{g} g + \sigma^{-}_{go}(o) \sigma^{-}_{gm_{2}}(m_{2}) \\ \dot{s} &= -\gamma_{s} s + \sigma^{+}_{sg}(g)\sigma^{-}_{sm_{1}}(m_{1})\\ \dot{z} &= -\gamma_{z} z + \sigma^{+}_{zs}(s) \sigma^{-}_{gm_{2}}(m_{2})\sigma^{-}_{zo}(o) \\ \dot{o} &= -\gamma_{o} o + \sigma^{-}_{oz} z \\ \dot{m}_{1} &= -\gamma_{m_{1}} m_{1} + \sigma^{-}_{m_{1}z}(z)\sigma^{-}_{m_{1}s}(s) \\ \dot{m}_{2} &= -\gamma_{m_{2}} m_{2} + \sigma^{-}_{m_{2}z}(z)\sigma^{-}_{m_{2}s}(s), \end{array} $$

where variables *g* (=[TGF *β*]), *s* (=[Snail1]), *z* (=[Zeb1]), *o* (=[Ovol2]), *m*_1_ (=[miR34a]) and *m*_2_ (=[miR200]) represent the indicated concentrations.

#### **Remarks 1**

Note that $\sigma ^{+}_{ij} (x)$ can be viewed as a limit of Hill functions $f^{+}_{n}(x) = L_{ij} + \frac {(U_{ij} -L_{ij}) x^{n}}{\theta _{ij}^{n} + x^{n}}$ as *n*→*∞*, and $\sigma ^{-}_{ij} (x)$ is a limit of Hill functions $f^{-}_{n}(x) = L_{ij} + (U_{ij} -L_{ij}) \frac {\theta _{ij}^{n} }{\theta _{ij}^{n} + x^{n}}$ as *n*→*∞*. This observation allows a translation between DSGRN model and Hill type model, with the exception of Hill coefficient *n*.

### Phase space and the state transition graph

The thresholds {*θ*_*ij*_} decompose the phase space [0,*∞*)^*n*^ into finitely many n-dimensional *cells* *κ*. In order to avoid degenerate cells, we assume that for all *j*≠*k*,

$$\theta_{ji} \neq \theta_{ki}, i = 1, \ldots, n.$$

Let *S*_*i*_:={0,…,*p*_*i*_} be the set of integers indexing the set of *p*_*i*_ outgoing edges and hence the set of thresholds of variable *x*_*i*_. We label any cell *κ* by an integer vector *s*=(*s*_1_,…,*s*_*n*_),*s*_*i*_∈*S*_*i*_, where *s*_*i*_ is the number of thresholds *θ*_*ji*_ below the *x*_*i*_ component of arbitrary *x*_*i*_∈*κ*. We call *s* a *state* of the vertex *i*. Then the state transition graph (STG) has the set of vertices

$$S := \prod_{i=1}^{n} S_{i}, $$

which is the set of labels of all cells *κ*.

Now assume that the parameters of (2) are fixed. Note that each *Λ*_*i*_ is constant for *x*∈*κ*, and so is *Λ*(*κ*):=(*Λ*_1_(*κ*),…,*Λ*_*n*_(*κ*)). If *Λ*(*κ*) is a constant then straightforward inspection of (2) shows that the solutions of (2) in *κ* will cross each of the *n*−1 dimensional boundaries of *κ* either in one or the other direction given a generic assumption

$$0 \neq -\gamma_{i}\theta_{ji} + \Lambda_{i}(\kappa)\quad \text{ for all} i,j, \kappa,$$

explained further in Remark 2. We now describe the construction of the state transition graph that reflects the dynamics of (2).

Consider cells *κ*,*κ*^′^, with state labels *s*,*s*^′^, that share an (*n*−1)-dimensional face *ϱ* in the *x*_*i*_ direction, and whose *x*_*i*_-coordinate value is *θ*_*ji*_. Then the edge is pointing from *s* to *s*^′^ if

- the *x*_*i*_-coordinate values of the points in *κ* are below *θ*_*ji*_ and −*γ*_*i*_*θ*_*ji*_+*Λ*_*i*_(*κ*)>0; or
- the *x*_*i*_-coordinate values of points in *κ* are above *θ*_*ji*_ and −*γ*_*i*_*θ*_*ji*_+*Λ*_*i*_(*κ*)<0.

#### **Remarks 2**

To achieve consistency in these rules so that for every pair of neighboring cells *κ*,*κ*^′^ there is an edge either *s*→*s*^′^, or *s*^′^→*s* we assume that

*A regulatory network does not admit negative self-regulation.*

It is easy to see that the inconsistency can only happen if the value of *Λ*_*i*_(*κ*)>*Λ*_*i*_(*κ*^′^) for *x*_*i*_<*y*_*i*_ for *x*∈*κ*,*y*∈*κ*^′^. This can only happen when node *i* negatively regulates itself.

If there is *κ* for which all edges from its neighboring cells are incoming, we assign a self-edge to its state *s*.

### Parameter graph

The parameter space of our system is the collection of degradation rates *γ*_*i*_, *i*=1,…,*n*, and triples (*U*_*ij*_,*L*_*ij*_,*θ*_*ij*_), one for each edge from *j* to *i* with *i*,*j*=1,…,*n*. Recall that *p*_*i*_ is the number of downstream genes of node *i* in the network. We denote by *o*_*i*_ a particular ordering of actuation thresholds $\theta _{j_{1}i} < \cdots < \theta _{j_{p_{i}}i}$ for the *p*_*i*_ out-edges of node *i*, and collect *o*_*i*_ for all nodes *i*=1,…,*n* of the network as

$$O := (o_{1},o_{2},\dots,o_{n}). $$

A nonempty region defined by a particular set *O* of orderings of actuation thresholds and a particular instantiations of the inequalities

$$ 0 < -\gamma_{i}\theta_{j_{k}i} + \Lambda_{i}(\kappa) \text{ or} 0 > -\gamma_{i}\theta_{j_{k}i} + \Lambda_{i}(\kappa), $$

one choice for every combination of *k*=1,…,*p*_*i*_ and*i*=1,…,*n*, and*κ*

is a parameter region. *Λ*_*i*_(*κ*) is defined in “Switching system” section. Each set of inequalities unambiguously determines the vector field (2) in each domain *κ* [26]. The collection of all parameter regions decomposes parameter space into a collection of open domains whose closure covers the parameter space.

**Fig. 8:**
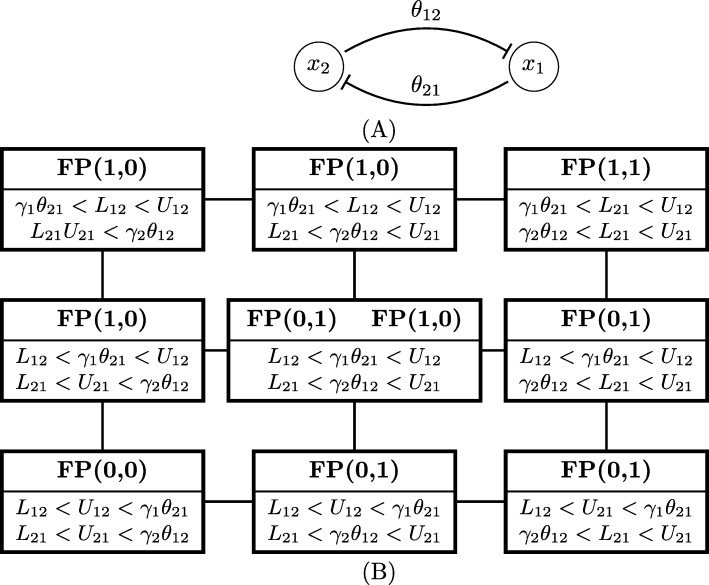
Parameter graph for toggle switch. **a** Toggle switch network. **b** Parameter graph for the toggle switch has 9 parameter nodes. Each parameter node correspond to a domain in the parameter space given by the inequalities listed in the node. Morse graph description is above the line in each node. FP (a,b) denotes a stable fixed point in the domain (a,b), where a, b are integers. The node exhibiting bistability is in the center

In Fig. 8b, we show the parameter graph for the toggle switch network in Fig. 8a. There is one threshold for each of the two variables in phase space, which is divided to four domains. Therefore, the state transition graph has four nodes. The combinations of switching functions *Λ*_*i*_ can each take two values: *Λ*_1_∈{*L*_12_,*U*_12_} and *Λ*_2_∈{*L*_21_,*U*_21_}. The parameter graph for the toggle switch has 9 parameter nodes. Each parameter node corresponds to a region in parameter space given by the inequalities listed in the node. The Morse graph description is above the line in each node. FP(a,b) denotes a stable fixed point in the domain (a,b), where a, b are integers. The node exhibiting bistability is in the center.

The parameter graph is a graph where each vertex, which is called a *parameter node*, corresponds to a nonempty parameter region, and there is an edge between two parameter nodes if and only if the corresponding regions share a co-dimension one boundary. This means that the defining set of inequalities differ in a sign of exactly one inequality. This graph captures all the different patterns of actuation that are compatible with the network structure. Details about how to construct a parameter graph can be found in [26].

## Data Availability

The procedure for generating the simulations analyzed during the current study are available in the 2020-Multistability-EMT code repository 10.5281/zenodo.3633343.

## Abbreviations

DSGRN: Dynamic signatures generated by regulatory networks
E state: Epithelial state
E/M state: Partially epithelial and partially mesenchymal intermediate states
EMT: Epithelial-to-mesenchymal transition
M state: Mesenchymal state
MET: Mesenchymal-to-epitelial transition
ODE: Ordinary differential equation
STG: State transition graph

## References

[1] Nakaya Y, Sheng G (2013). EMT in developmental morphogenesis. Cancer Lett.

[2] Thiery J, Acloque H, Huang R, Nieto M (2009). Epithelial-mesenchymal transitions in development and disease. Cell.

[3] Arnoux V, Côme C, Kusewitt D, Hudson L, Savagner P (2005). Cutaneous wound reepithelialization. Rise and fall of epithelial phenotype.

[4] Savagner P, Arnoux V (2009). Epithelio-mesenchymal transition and cutaneous wound healing. Bull Acad Natl Med.

[5] Dongre A, Weinberg R (2019). New insights into the mechanisms of epithelial-mesenchymal transition and implications for cancer. Nat Rev Mol Cell Biol.

[6] Mani S, W G, Liao M. -J., Eaton E, Ayyanan A, e.a. (2008). The epithelial-mesenchymal transition generates cells with properties of stem cells. Cell.

[7] Singh A, Settleman J (2010). EMT, cancer stem cells and drug resistance: an emerging axis of evil in the war on cancer. Oncogene.

[8] Gajewski T, Schreiber H, Fu Y (2013). Innate and adaptive immune cells in the tumor microenvironment. Nat Immunol.

[9] Kerkar S, Restifo N (2012). Cellular constituents of immune escape within the tumor microenvironment. Cancer Res.

[10] Aceto N, Toner M, Maheswaran S, Haber D (2015). En route to metastasis: Circulating tumor cell clusters and epithelial- to-mesenchymal transition. Trends Cancer.

[11] Hong T, Watanabe K, Ha Ta C, Villarreal-Ponce A, Nie Q, Dai X (2015). An Ovol2-Zeb1 mutual inhibitory circuit governs bidirectional and multi-step transition between epithelial and mesenchymal states. PLoS Comput Biol.

[12] Zhang J, Tian X, Zhang H, Teng Y, Li R, Bai F, Elankumaran S, Xing J (2014). TGF- *β*-induced epithelial-to-mesenchymal transition proceeds through stepwise activation of multiple feedback loops. Sci Signal.

[13] Grosse-Wilde A, Fouquier d’Herouei A, McIntosh E, Ertaylan G, Skupin A, Kuestner R, del Sol A, Walters K-A, Huang S (2015). Stemness of the hybrid epithelial/mesenchymal state in breast cancer and its association with poor survival. PLoS One.

[14] Pastushenko I, Brisebarre A, Sifrim A, Fioramonti M, Revenco T, Boumahdi S, Van Keymeulen A, Brown D, Moers V, Lemaire S, De Clercq S, Minguijón E, Balsat C, Sokolow Y, Dubois C, De Cock F, Scozzaro S, Sopena F, Lanas A, D’Haene N, Salmon I, Marine J, Voet T, Sotiropoulou P, Blanpain C (2018). Identification of the tumour transition states occurring during emt. Nature.

[15] Andriani F, Bertolini G, Facchinetti F, Baldoli E, Moro M, Casalini P, Caserini R, Milione M, Leone G, Pelosi G, Pastorino U, Sozzi G, Roz L (2016). Conversion to stem-cell state in response to microenvironmental cues is regulated by balance between epithelial and mesenchymal features in lung cancer cells. Mol Oncol.

[16] Yu M, Bardia A, Wittner B, Stott S, Smas M, Ting D, Isakoff S, Ciciliano J, Wells M, Shah A, Concannon K, Donaldson M, Sequist L (2013). Circulating breast tumor cells exhibit dynamic changes in epithelial and mesenchymal composition. Science.

[17] Tam W, Weinberg R (2013). The epigenetics of epithelial-mesenchymal plasticity in cancer. Nat Med.

[18] Savagner P (2015). Epithelial-mesenchymal transitions: from cell plasticity to concept elasticity. Curr Top Dev Biol.

[19] Tian X-J, Zhang H, Xing J (2013). Coupled reversible and irreversible bistable switches underlying TGF *β*-induced epithelial to mesenchymal transition. Biophys J.

[20] Lu M, Jolly M, Levine H, Onuchic J, Ben-Jacob E (2013). MicroRNA-based regulation of epithelial hybrid- mesenchymal fate determination. Proc Natl Acad Sci USA.

[21] Jolly M, Tripathi S, Jia D, Mooney S, Celiktas M, Hanash S, Mani S, Pienta K, Ben-Jacob E, Levine H (2016). Stability of the hybrid epithelial/mesenchymal phenotype. Oncotarget.

[22] Chaffer C, Marjanovic N, Lee T, Bell G, Kleer Cea (2013). Poised chromatin at the ZEB1 promoter enables breast cancer cell plasticity and enhances tumorigenicity. Cell.

[23] Guo D, Xu B, Zhang X, Dong M (2012). Cancer stem-like side population cells in the human naso-pharyngeal carcinoma cell line cne-2 possess epithelial mesenchymal transition properties in association with metastasis. Oncol Rep.

[24] Huang B, Lu M, Jia D, Ben-Jacob E, Levine H, Onuchic J (2017). Interrogating the topological robustness of gene regulatory circuits by randomization. PLoS Comput Biol.

[25] Font-Closa F, Zapperia S, La Portac CAM (2018). Topography of epithelial-mesenchymal plasticity. PNAS.

[26] Cummins B, Gedeon T, Harker S, Mischaikow K, Mok K (2016). Combinatorial Representation of Parameter Space for Switching Systems. SIAM J Appl Dyn Syst.

[27] Gedeon T, Cummins B, Harker S, Mischaikow K (2018). Identifying robust hysteresis in networks. PLoS Comput Bio.

[28] Cummins B, Gedeon T, Harker S, Mischaikow K (2018). Model rejection and parameter reduction via time series. SIAM J Appl Dyn Syst.

[29] Cummins B, Gedeon T, Harker S, Mischaikow K, Koeppl JFH (2017). Database of dynamic signatures generated by regulatory networks (DSGRN). Computational Methods in Systems Biology - 2017, Chap. 19.

[30] Huttinga Z, Cummins B, Gedeon T, Mischaikow K (2018). Global dynamics for switching systems and their extensions by linear differential equations. Phys D Nonlinear Phenom.

[31] Crawford-Kahrl P, Cummins B, Gedeon T (2019). Comparison of two combinatorial models of global network dynamics. SIAM J Appl Dyn Syst.

[32] Glass L, Kauffman Sa (1972). Co-operative components, spatial localization and oscillatory cellular dynamics. J Theor Biol.

[33] Glass L, Kauffman Sa (1973). The logical analysis of continuous, non-linear biochemical control networks. J Theor Biol.

[34] Jia D, Jolly MK, Boareto M, Parsana P, Mooney SM, Pienta KJ, Levine H, Ben-Jacob E (2015). OVOL guides the epithelial-hybrid-mesenchymal transition. Oncotarget.

[35] Newhouse SE (1979). The abundance of wild hyperbolic sets and nonsmooth stable sets for diffeomorphisms. Inst Hautes Études Sci Publ Math.

[36] Palis J, Takens F (1993). Hyperbolicity and Sensitive Chaotic Dynamics at Homoclinic Bifurcations. Cambridge Studies in Advanced Mathematics, vol. 35.

[37] Conley CC (1978). Isolated Invariant Sets and the Morse Index. Regional conference series in mathematics.

[38] Mischaikow K (2002). Topological techniques for efficient rigorous computation in dynamics. Acta Numer.

[39] Mischaikow K, Mrozek M. Conley index. In: Handbook of Dynamical Systems, Vol. 2. Amsterdam: North-Holland: 2002. p. 393–460. https://doi.org/10.1016/S1874-575X(02)80030-3. http://dx.doi.org.proxy.libraries.rutgers.edu/10.1016/S1874-575X(02)80030-3.

[40] Kalies W, Mischaikow K, Vandervorst R (2014). Lattice structures for attractors I. J Comp Dyn.

[41] Kalies WD, Mischaikow K, Vandervorst RCAM. Lattice Structures for Attractors II. Found Comput Math. 2015. 10.1007/s10208-015-9272-x.

